# Effects of Molecular Weight Distribution on the Thermal–Mechanical Performance and Recycling of CO_2_‑Derived Poly(cyclopentene carbonate)

**DOI:** 10.1021/acs.macromol.5c03136

**Published:** 2026-01-12

**Authors:** Balázs Striker, Alexander R. Craze, Kam C. Poon, Thomas M. McGuire, Kristian L. Mears, Charlotte K. Williams

**Affiliations:** Department of Chemistry, 6396 University of Oxford, Oxford Ox1 3ta, United Kingdom

## Abstract

Poly­(cyclopentene carbonate) (PCPC) is a recyclable, CO_2_-derived thermoplastic with high tensile strength and low entanglement molecular weight. Such CO_2_-derived polycarbonates typically show bimodal molecular weight distributions, but how these distributions influence their properties is not yet understood. Here, the tensile, mechanical, thermal, and recycling properties are investigated for PCPC samples with different bimodal molecular weight distributions. Samples with high molecular weights (*M*_n_ ∼ 81 kg mol^–1^) and narrow-gap bimodality, showing a relative 1:2 chain length distributions, are prepared using variable alcohol:diol ratios. These narrow-gap bimodality PCPC samples all show the same high tensile strength (σ_max_ ∼ 60 MPa) and glass transition temperature (*T*_g,∞_ = 88 °C). A second series features different relative amounts of high molecular weight PCPC (*M*_n_ = 76 kg mol^–1^) blended with low molecular weight samples (*M*_n_ = 9 or 16 kg mol^–1^). These wide-gap bimodality PCPC samples generally show compromised thermal and mechanical performance, with properties only being retained when low amounts of chains with molecular weights above chain entanglement are added. All the high-*M*_n_ PCPC samples are rapidly depolymerized, using neat polymer-catalyst blends, to produce cyclopentene oxide and carbon dioxide, regardless of molecular weight distribution. Complete conversion to epoxide (CPO) and CO_2_ is achieved in <15 min at 140 °C (1:300 catalyst:PCPC repeat unit).

## Graphical abstract

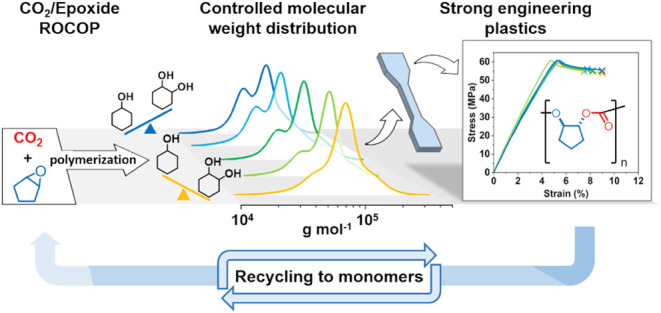

## Introduction

Making plastics more sustainable is essential for both economic and environmental reasons. Key targets to achieve greater sustainability include maximizing the use of renewable feedstocks and producing materials that can undergo energy- and material-efficient recycling after use.^1,2^ Only by implementing both strategies can the polymer manufacturing industry’s significant greenhouse gas emissions be mitigated.^3–5^ Using captured CO_2_ as a cheap and abundant carbon source is attractive since it obviates the land-use, biodiversity loss, and nitrogen cycle impacts that can be associated with using biomass.^6^ One of the most advanced CO_2_ utilization technologies is the ring-opening copolymerization (ROCOP) of epoxides and CO_2_.^7–9^ When using bicyclic epoxides, like cyclopentene oxide (CPO) or cyclohexene oxide (CHO), the products are amorphous polycarbonates with high glass transition temperatures (*T*_g_) and high tensile strengths, making them promising as engineering plastics.^10,11^ For example, poly­(cyclohexene carbonate) (PCHC) has a tensile strength of σ_max_ = 40–43 MPa^12,13^ and *T*_g_ = 105–126 °C,^12–14^ with a *T*_g,∞_ = 126 °C.^15^ Its major drawback is high brittleness, with an elongation at break of <3%.^10,12–14^ Another barrier to accessing high-performance PCHC is its very high entanglement molecular weight (*M*_e_) of 56 kg mol^–1^, determined by oscillatory rheology,^10^ meaning optimum mechanical performance requires PCHC molecular weights several multiples (4–7 times) higher. Achieving such high molecular weights is very demanding synthetically, and processing these materials is expected to be very challenging due to their high viscosity.^10^ On the other hand, poly­(cyclopentene carbonate), PCPC, also shows a high tensile strength, σ_max_ = 59 MPa, and slightly higher elongation at break, *ε*_b_ = 7%.^10^ In contrast, its entanglement molecular weight and viscosity are an order of magnitude lower than those of PCHC, with PCPC *M*_e_ = 4.0–4.9 kg mol^–1^; as such it is expected to be a better choice for engineering plastics application development.^10^ Terpolymers of PCPC–PCHC show interesting trends in *M*_e_ and better toughness than PCHC.^13^ While these preliminary studies suggest that a more accessible range of PCPC molecular weights might show good material performances, it is not yet clear how molecular weight distributions (MWDs) affect sample physical properties. Understanding the relationships between both molecular weight and its distribution with material strength, toughness, and processability is essential for any material design or use.^16,17^ In the case of CO_2_-ROCOP-synthesized materials, where bimodal MWDs are very common, and can often only be avoided at the cost of excessive purification^18^ or the use of specialist catalysts,^10^ this issue is even more relevant.

Here, we seek to investigate the influences of different types of molecular weight distributions on PCPC properties by producing samples with variable molecular weights and distributions, including those that mimic issues that may arise in the larger-scale production of CO_2_-based polycarbonates. In these CO_2_/epoxide ROCOP reactions, the most common contaminant is water, which is particularly difficult to completely remove from CO_2_.^18^ Wu and Darensbourg showed that, for many catalysts, trace amounts of water rapidly react with the epoxide to form the corresponding diol, which subsequently functions as a chain transfer agent (CTA) in the catalysis. Chain transfer agents reversibly displace growing polymer chains from the catalyst and initiate new chains by reacting with the catalytic alkoxide intermediate in an equilibrium.^19^ It is well-known that CTAs decrease the number-average molecular weight *M*_n_ of the resulting polymer and influence MWDs.^18,20^ Diols lead to the formation of α,ω-dihydroxyl end-capped chains, whereas alcohols or catalyst-initiating ligands typically form only α-hydroxyl end-capped chains.^20–22^ When such dihydroxyl telechelic chains are desired, for example, in ABA-block polymer synthesis, adding water to the reaction can serve as a convenient and cheap CTA.^19,22^ While the effect of residual moisture in the epoxide is expected to be instantaneous,^19^ a wet CO_2_-stream may introduce water steadily throughout the reaction, particularly in any continuous gas addition which would be essential at larger-scale. Considering the amounts of water which might be present, it is relevant that captured CO_2_ usually contains 20–650 ppm by volume (ppmv) water,^23^ and carbon capture and storage (CCS) specification proposals recommend limiting water content to 100–500 ppmv.^23–25^ These water contamination levels are used to establish the comparable alcohol/diol loadings used in this study (8 × 10^–4^ equivalents per monomer, i.e., 800 ppm). Note that these CCS proposals consider contaminant levels in light of CO_2_/water phase behavior and pipeline corrosion rather than chemical utilization, but any CCU scenario would need to account for similar contamination levels.^25,26^ Many catalysts are not very tolerant to water, and in these cases, CO_2_ drying is essential. For example, Jia et al. studied the effects of different grades of CO_2_-drying showing a progressive increase in *M*_n_ and an increased share of catalyst-initiated PCHC chains compared to the water-derived α,ω-dihydroxyl end-capped ones with more efficient drying.^18^ In the experiments, water was removed by passing the CO_2_ through highly reactive and pyrophoric triisobutylaluminum prior to the reaction—such conditions enabled the synthesis of monomodal, very high molecular weight PCHC. The PCHC physical properties were not reported, and thus, it is unclear whether these purification steps provide a superior material. Water (and diols formed from it) are not the only contaminants that may act as chain transfer agents. Epoxides may also carry monofunctional initiator alcohols, for example, cyclohexanol.^27,28^ Some catalysts are also proposed to react by modified MPVO (Meerwein- Ponndorf-Verley-Oppenauer) reactions to transform cyclohexene oxide into cyclohexanol.^29^ Therefore, it is important to account for the influences on the product molecular weights and distributions of catalyst-initiating groups, alcohols, and diols (formed from residual water).

The best catalysts must show good tolerance to hydroxyl impurities, high turnover numbers (productivity), high turnover frequencies (activities), and good polycarbonate selectivity. Several excellent catalyst classes have been developed for CHO/CO_2_ ROCOP, including cobalt­(III)­(salen)­(X)/PPNX complexes (where X = halide/carboxylate, and PPN = phosphiniminium),^30^ porphyrin-supported Al-catalysts,^31^ multinuclear L’lanthanide­[Co]_3_(OAc)_2_,^32^ β-diiminate-bound monometallic,^33^ dimeric,^34^ and tethered dizinc complexes,^35,36^ as well as LZn­(II)­Mg­(II)­(OAc)_2_ and LCo­(II)­Mg­(II)­(OAc)_2_ complexes (L or L’ are macrocyclic ancillary ligands).^37,38^ The latter heterodinuclear catalysts both show high tolerance to water or other protic compounds and operate effectively at high temperatures.^22,37,39,40^ Borane-ammonium or phosphonium catalysts also show good activities and selectivity for CHO/CO_2_ ROCOP.^18,41,42^ Most prior catalysis investigations applied CHO/CO_2_ ROCOP, but the analogous five-membered ring epoxide CPO/CO_2_ ROCOP is often considerably slower; therefore, fast catalysis is even more important for PCPC production.^10,43,44^ Several of these catalysts are reported to produce PCPC with high selectivity and good activity,^13,42,45^ including the LCo­(II)­Mg­(II)­(OAc)_2_ catalyst, which is used in this manuscript.^10,44,46^ In this field, most catalysts feature initiating ligands, e.g., acetate or halide, which result in polycarbonates showing bimodal MWDs due to chains being initiated by both the catalyst and diol (formed from residual water). Our team tackled the molecular weight distribution challenge by using an organometallic LCo­(II)­Mg­(II)­(C_6_F_5_)_2_ catalyst for CPO/CO_2_ ROCOP, which retained the high activity and enabled initiation only from the added diol, thereby selectively producing α,ω-dihydroxyl end-capped PCPC with monomodal MWD, even at high *M*_*n*_ = 114 kg mol^–1^.^10^ However, the synthesis of such noninitiating catalysts is rather challenging and requires anaerobic (Schlenk line) handling protocols. It would be desirable to use the air-stable, easy-to-prepare LCo­(II)­Mg­(II)­(OAc)_2_ catalyst and to understand whether the resulting PCPC bimodal molecular weight distributions diminish material properties.

PCPC has also attracted attention because of its low-energy recyclability, including mechanical reprocessing^10^ and chemical depolymerization to monomers (CO_2_, CPO).^42,47^ Previously, we showed that LCo­(II)­Mg­(II)­(OAc)_2_ showed high depolymerization activity in neat polycarbonate films, including PCPC.^48,49^ Chemical recycling to monomers is considered a key method to enable a circular polymer economy.^50^ In this study, we seek to understand whether the same catalyzed PCPC depolymerization rates are influenced by sample MWD.

## Results and Discussion

As mentioned, water contaminating the CO_2_ forms diols, which are chain transfer agents in CO_2_/epoxide ROCOP, and the resulting polycarbonates show bimodal molecular weight distributions with a characteristic 1:2 relative chain length relationship, here referred to as “narrow-gap” bimodality (Scheme 1).^18,37^ On the other hand, moisture might be introduced at later polymerization stages, resulting in polycarbonates comprising both high and low molecular weight chains, here referred to as “wide-gap” bimodality. The latter distribution is hard to reproduce in small-scale reactor setups; hence, these wide-gap distributions are modeled by forming blends of low- and high-*M*_n_ PCPC samples. To form the blends, monomodal PCPC samples are needed with molecular weights ranging from low (*M*_n_ <10 kg mol^–1^) to high (*M*_n_ ∼80 kg mol^–1^). To prepare all samples, the LCo­(II)­Mg­(II)­(OAc)_2_ catalyst was selected for its high turnover frequency since cyclopentene oxide is expected to polymerize slower than cyclohexene oxide.^51,52^ While acetate initiation can lead to bimodal MWDs,^10^ monomodal distributions should occur when using high loadings of chain transfer agent (since the catalyst-initiated chains are effectively suppressed).

**Scheme 1:**
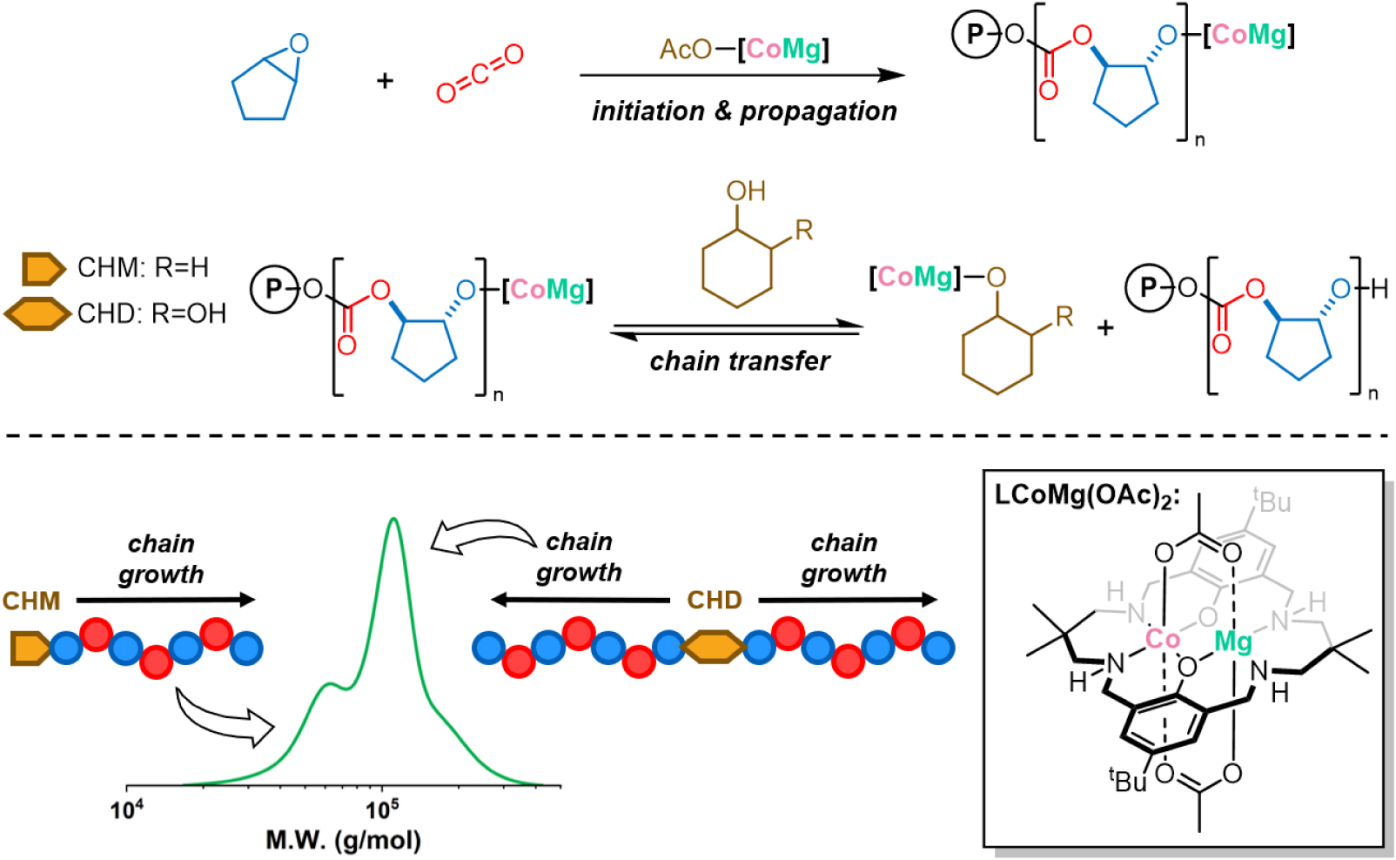
Illustration of Cyclopentene Oxide (CPO)/CO_2_ ROCOP, Using the LCo­(II)­Mg­(II)­(OAc)_2_ Catalyst and Chain Transfer Agents (CTAs), to Produce Poly­(cyclopentene carbonate) (PCPC)^a^

a Also shown are the structures of the two chain transfer agents cyclohexanol (CHM) and 1,2-cyclohexene diol (CHD) used in this work, and the resulting bimodal molecular weight distributions (the mechanisms for initiation and propagation are shown in **Scheme S1**)

Thus, a series of polymerization experiments were conducted with a 1:10,000 LCo­(II)­Mg­(II)­(OAc)_2_:CPO loading, using varying amounts of 1,2-cyclohexene diol (CHD) (Table 1). Reactions conducted with 50 equivalents of diol per catalyst led to PCPC showing effectively monomodal molecular weight distributions, i.e., the majority of chains are initiated from the diol. Terminating the reaction prematurely (57% CPO conversion) yielded **PCPC-9** (*M*_n_ = 9.3 kg mol^–1^). Stopping the reaction at 89% CPO conversion achieves *M*_n_ = 16 kg mol^–1^ **PCPC-16**, with the higher molecular weight directly proportional to epoxide conversion, as expected for a controlled polymerization. Reducing the diol loading to 23 equiv per catalyst gave **PCPC-37** (*M*_n_ = 37 kg mol^–1^). Using even lower catalyst loading relative to CPO, and just 8 equiv of CHD, led to high-*M*_n_ **PCPC-76**. In this case, the major peak was accompanied by a lower molecular weight shoulder, as observed by gel permeation chromatography (GPC) (Figure 1a). The shoulder peak is assigned to catalyst (acetate)-initiated chains, while the major peak is assigned to chains initiated by the diol. All samples showed similar dispersity, 1.2 < *Đ* < 1.3. The catalytic turnover frequencies (TOF) ranged between 120 and 460 h^–1^, which are at the upper end of values for CPO/CO_2_ ROCOP (Table S1) and match those for the previously reported LCo­(II)­Mg­(II)­(C_6_F_5_)_2_ organometallic catalyst.^10^ Importantly, the LCo­(II)­Mg­(II)­(OAc)_2_ catalyst proved tolerant to high monomer loadings up to 1:20,000, resulting in excellent productivity, with a TON of 18,000.

**Table 1:** Data for Monomodal PCPC Syntheses Targeting a Range of *M*_n_ a

[cat]:[CHD]:[CPO]	TON^[^ ^b^ ^]^	Conv.^[^ ^c^ ^]^	Selec.^[^ ^d^ ^]^	*M*_n,GPC_^[^ ^e^ ^]^(kg mol^–1^)	*Đ* ^[^ ^e^ ^]^ (*M*_w_/*M*_n_)	*T*_g_^[^ ^f^ ^]^(°C)
1:8:20,000	18,000	90%	98%	76	1.24	87
1:23:10,000	9400	94%	96%	37	1.24	86
1:50:10,000	8900	89%	91%	16	1.27	82
1:50:10,000	5700	57%	98%	9.3	1.21	81

a CPO/CO_2_ ROCOP conditions: 100 °C, 20 bar CO_2_, toluene, [CPO]_0_ = 6 M.

b TON = epoxide conversion × [CPO]_i_/[catalyst].

c ([PCPC] + [cyclic carbonate])/([CPO] + [PCPC] + [cyclic carbonate]), determined by ^1^H NMR spectroscopy.

d [PCPC]/([PCPC]+[cyclic carbonate]), determined by ^1^H NMR spectroscopy.

e Determined by GPC (THF eluent, calibrated by polystyrene standard).

f Determined by DSC from the second heating cycle.

**Figure 1:**
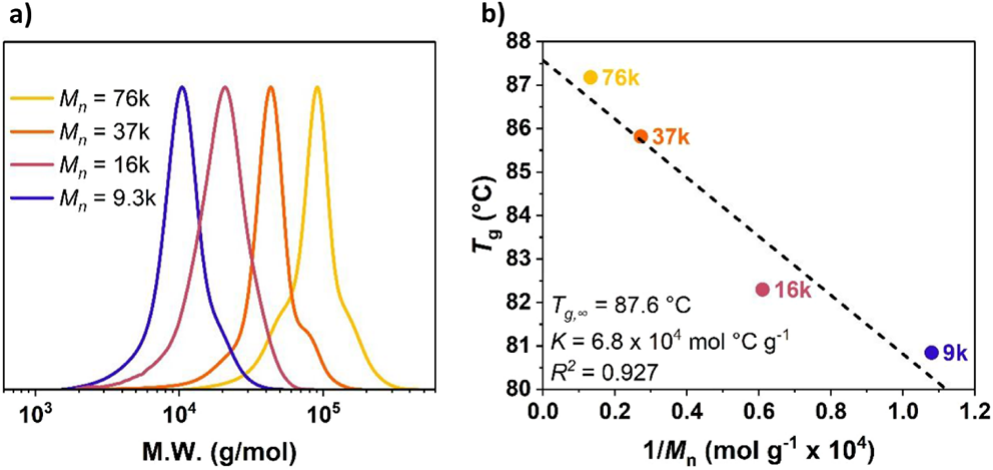
(a) GPC chromatograms of the majority monomodal molecular weight distribution of the PCPC series. (b) Glass transition temperature values of the same polymers as a function of inverse *M*_n_, fitted against the Flory–Fox equation (Equation S2).^53^ *T*_g_ values were determined by DSC (second heating cycle).

The thermal properties of the four PCPC samples were measured by differential scanning calorimetry (DSC, Figure S16). All of the polycarbonates are amorphous and show a single glass transition temperature in the range of *T*_g_ = 81–87 °C. The *T*_g_ values are in agreement with the Flory–Fox equation,^53^ describing the relationship between *M*_n_ and *T*_g_ (Equation S2). Thus, plots of the *T*_g_ values against 1/*M*_n_ show a linear trend, and fits result in *T*_g,∞_ = 88 °C as the maximum glass transition temperature at infinite chain length (Figure 1b). All samples were analyzed by thermogravimetric analysis (TGA) and showed *T*_d,5%_ = 250–270 °C (Figure S17).

The presence of both mono- and di-initiating species (CTAs) at the start of the ROCOP reaction will result in a “narrow-gap” bimodal MWD.^19,29^ To investigate this situation, we conducted catalysis with both an alcohol and a diol added to the CPO/CO_2_ ROCOP. While the expected impurities in CPO would be cyclopentanol and its diol, in these experiments, cyclohexanol (CHM) and cyclohexane diol (CHD) were used due to price and availability considerations, under the assumption of very similar reactivities. Using a 1:8 catalyst (monoinitiator):CHD (diinitiator) ratio, the resulting **PCPC-76** was effectively monomodal. For the rest of the PCPC series, progressively more alcohol, CHM, was added to the starting mixture, keeping the total concentration of CHM + CHD at a constant 8 equivalents per catalyst. Increasing monoinitiator concentration should make the resulting molecular weight distribution more bimodal while retaining a similar overall *M*_n_, since there should be an approximately constant concentration of chain transfer agents in all experiments. All polymerizations achieved high conversions (>85%), high polymer selectivity (>97%), very high productivity (∼18,000), and good activity (Table 2).

**Table 2:** Data for the Production of PCPC with Narrow-Gap Bimodal MWD^a^

[cat]:[CHM]:[CHD]:[CPO]	TON^[^ ^b^ ^]^	Conv.^[^ ^c^ ^]^	Selec.^[^ ^d^ ^]^	*M*_n_^[^ ^e^ ^]^ (kg mol^–1^)	*Đ* ^[^ ^e^ ^]^ (*M*_w_/*M*_n_)
1:0:8:20,000	18,000	90%	98%	76	1.24
1:2:6:20,000	18,400	92%	98%	89	1.19
1:4:4:20,000	17,000	85%	97%	88	1.23
1:6:2:20,000	17,800	89%	98%	71	1.39
1:8:0:20,000	17,600	88%	98%	81	1.28

a CPO/CO_2_ ROCOP conditions: 100 °C, 20 bar CO_2_, toluene, [CPO]_0_ = 6 M.

b TON = epoxide conversion×[CPO]_i_/[catalyst].

c ([PCPC] + [cyclic carbonate])/([CPO] + [PCPC] + [cyclic carbonate]), determined by ^1^H NMR spectroscopy.

d [PCPC]/([PCPC]+[cyclic carbonate]), determined by ^1^H NMR spectroscopy.

e Determined by GPC (THF eluent, calibrated by polystyrene standards).

The PCPC sample molecular weights, determined by GPC, ranged from 76 to 89 kg mol^–1^ and displayed an increasing extent of narrow-gap bimodality in the MWD (Figure 2). This finding is fully consistent with all hydroxyl groups reacting equivalently as initiators and chain transfer agents, resulting in uniform rates of chain growth on all active chain ends (Scheme 1).^54^ There are two important consequences: as the CHD-initiated chains propagate from two hydroxyl end groups, the resulting PCPC chains will be twice as long as the monoinitiated chains end-capped by CHM.^55^ In all these polymers, there is both a lower molecular weight peak at *M*_n_ ∼60 kg mol^–1^ and a higher one at *M*_n_ ∼110 kg mol^–1^, showing an ∼1:2 MW ratio (Figure 2a). Second, while CHD-initiated chains grow to double the length of the CHM-initiated ones in the same system, the number-average molecular weight (*M*_n_) should be controlled by the overall number of chains per monomer, regardless of the number of hydroxyls on each initiator. In these types of controlled polymerization catalysis, it is assumed that all chain initiation reactions occur at equal rates and that chain transfer is significantly faster than propagation, regardless of the hydroxyl environment. Thus, at constant CHM + CHD concentration, the resulting *M*_n,GPC_ was similar within the series (within 10% of an average of ∼81 kg mol^–1^) regardless of the CHM:CHD ratio. A clear trend in the relative intensity of the two peaks was observed to reflect the relative amounts of the two alcohols (CHM:CHD). Accordingly, the lower molecular weight peak (corresponding to CHM- and catalyst-initiated chains) increases in intensity with increased CHM loading. The “double-weight” higher peak remained prominent within the series, however, even when no di-initiating CHD was added. This finding is, at first, confusing, but likely arises due to the presence of residual moisture in the CO_2_-stream (or residual diol in the epoxide), which is both very common in epoxide/CO_2_ ROCOP.^18,56,57^ As complete removal of chain transfer agents from the monomers adds complexity and may be difficult at scale, it is important to understand how CTA-induced bimodality may affect material properties. To prepare the samples for processing, the polymers were purified by precipitation and filtration over silica to remove the catalyst. The polymers were then thoroughly dried at 110 °C under vacuum. The molecular weights and distributions (MWDs) were not changed significantly by this isolation process (Figures S5–S9).

**Figure 2:**
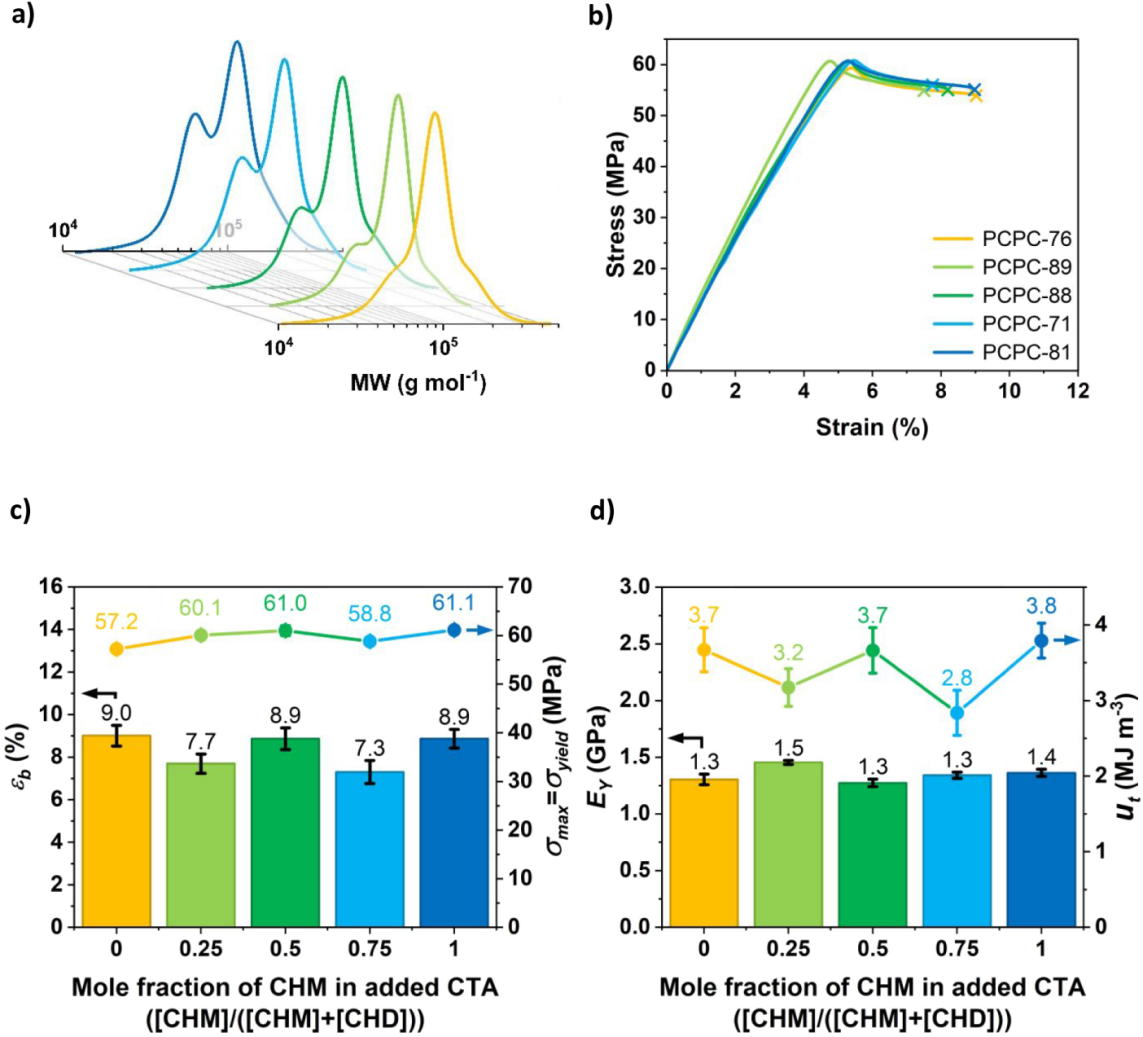
(a) GPC data for the narrow-gap bimodal PCPC series (for relative [CHM]:[CHD] data for each sample, see Table 2). (b) Representative tensile stress vs strain data for the narrow-gap bimodal PCPC series. Tensile testing was carried out at a 10 mm min^–1^ crosshead velocity (see SI for details). (c) Chart showing how the elongation at break and maximum tensile strength (equal to yield stress) of the PCPC series correlate with the relative [CHM] per total added CTA in synthesis. (d) Chart showing how the Young’s moduli and tensile toughness of the series correlate with the relative [CHM] per total added CTA in synthesis. The Young’s moduli data were obtained from the stress–strain gradient at low strains, and tensile toughness was obtained from the area under the stress–strain curves.

Thermal properties of the purified polymers were assessed by differential scanning calorimetry (DSC) and thermogravimetric analysis (TGA). The narrow-gap bimodality PCPC series all show very similar glass transition temperatures, with *T*_g_ = 87–88 °C (Table S3). Thus, in this high molecular weight regime, the thermal properties are unaffected by the sample MWD. All polymers exhibited good thermal stability, with *T*_d,5_= 245–271 °C (Figure S19).

ISO 527–2 type 5B tensile specimens were prepared from compression-molded sheets of each PCPC sample, and uniaxial tensile testing measurements were carried out (Figure 2b and Table S3). All PCPC samples displayed similar tensile properties, with little or no significant difference in tensile strength at σ_max_ ∼60 MPa and elongation at break *ε*_b_ ∼8%. These values closely match the monomodal high-*M*_n_ PCPC properties reported by Rosetto et al., where samples were prepared with the organometallic LCo­(II)­Mg­(II)­(C_6_F_5_)_2_ catalyst.^10^ In all cases, PCPC shows the characteristic deformation of glassy polymers, featuring a yield point and a short elongation phase before breaking. These results reinforce the potential of PCPC as a high-strength engineering plastic. All samples are brittle, with similar Young’s moduli and tensile toughness. It is interesting to note that narrow-gap bimodality does not compromise the mechanical performance, presumably as long as the molecular weight exceeds the critical value. This finding is probably quite significant, since achieving PCPC samples with both high molecular weights and monomodal distributions requires extensive monomer purification and/or noninitiating catalysts,^10,18^ thus tolerance to bimodality may simplify the process and broaden the catalyst choice, as long as the overall target *M*_n_ is met.

To evaluate any differences in sample processability, variable temperature oscillatory shear rheology experiments were performed on samples at the two extremes of modality, **PCPC-76** and **PCPC-81**. The samples both show very similar rheological behavior (Figures S36–S41). Glass transition temperatures, as determined by the tan­(δ) peak in the temperature ramps, were *T*_g,rheo_ = 98 °C. The *T*_g_ values obtained *via* this method were about 10 °C higher than those measured by DSC, which is in agreement with previous investigations of CO_2_-based polycarbonates (including PCPC).^10^ The terminal regime crossover temperatures, where storage modulus (*G’*) equals loss modulus (*G”*), were also similar, *T*_cross_ = 153 °C for **PCPC-76** and 159 °C for the bimodal **PCPC-81** (obtained at 1 Hz oscillatory frequency). Complex viscosities were calculated over the measured temperature range, with the two samples being very similar. At 140 °C (in the rubbery plateau regime) and 1 Hz frequency, η* = 45 kPa s was measured for **PCPC-76** and η* = 39 kPa s for **PCPC-81**. This minor reduction in viscosity may be attributed to the increased ratio of shorter chains.

As narrow-gap bimodality (polymers consisting of 1:2 molecular weight ratio chains) did not affect the mechanical performance of PCPC, investigations using wide-gap bimodality materials were then carried out. The wide-gap bimodality samples were prepared by mixing solutions of **PCPC-9** or **PCPC-16** with **PCPC-76**, accessing blends where the low- and high-MW PCPC components have relative *M*_n_ ratios of 1:8 and 1:5, respectively. **PCPC-37** was not used for blending, as it would lead to 1:2 *M*_n_ ratio bimodality, the same as the narrow-gap series. After thorough mixing, the blends were cast, dried, and processed in the same way as the narrow-gap PCPC samples. DSC analysis revealed that blending the low molecular weight **PCPC-9** sample into **PCPC-76** caused a gradual *T*_g_ decrease (Table 3). A linear correlation between the weight fraction of **PCPC-9** and the reciprocal of *T*_g_ was observed for these blends, suggesting the short chains are miscible with the longer chains of **PCPC-76** (Figure S22 and Equation S3).^58^ Interestingly, the same effect was not observed for the blends of **PCPC-16** with **PCPC-76**, where no significant change in glass transition temperatures was observed, despite the lower *T*_g_ of **PCPC-16**. The difference in behavior may arise from different chain entanglements: the chains of **PCPC-9** are below the entanglement limit and, as such, behave differently from high-weight PCPC (while remaining miscible). Rheological experiments suggest the lack of this entanglement since the frequency response of **PCPC-9** lacks the characteristic entangled rubbery plateau, unlike that of **PCPC-76** (Figures S33 vs S30). This is also reflected in the high brittleness of the material, which is easily shattered by simple manual handling. **PCPC-16**, on the other hand, has chains significantly above *M*_e_, and thus should entangle with the chains of **PCPC-76** without affecting its overall thermal properties, similarly to the narrow-gap bimodal PCPC entries, where *T*_g_ was not affected by the MWD either.

**Table 3:** Data for the Thermal and Tensile Properties of Wide-Gap Bimodal PCPC Samples, Where the Major Component is **PCPC-76**

Low-*M*_n_ PCPC content		*T*_g_ (°C)	*σ*_max_^[^ ^a^ ^]^ (MPa)	*ε*_b_^[^ ^b^ ^]^ (%)	*E*_Y_^[^ ^c^ ^]^ (GPa)	*U*_t_^[^ ^d^ ^]^ (MJ m^–3^)
	0%	87	57.2 ± 0.6	9.0 ± 0.5	1.30 ± 0.05	3.7 ± 0.3
						
	10%	86	52.2 ± 0.5	5.9 ± 0.3	1.33 ± 0.05	2.0 ± 0.1
**PCPC-9.3**	30%	86	50.4 ± 0.9	6.4 ± 0.4	1.38 ± 0.02	2.2 ± 0.2
	50%	84	47.5 ± 0.6	4.1 ± 0.1	1.44 ± 0.03	1.1 ± 0.1
	10%	88	56.6 ± 0.3	9.1 ± 0.7	1.33 ± 0.02	3.7 ± 0.4
						
**PCPC-16**	30%	87	50.3 ± 0.5	6.4 ± 0.1	1.30 ± 0.02	2.1 ± 0.0
	50%	87	51.5 ± 0.7	5.7 ± 0.1	1.46 ± 0.03	1.9 ± 0.1

a Maximum tensile strength, coinciding with yield stress.

b Elongation at break.

c Young’s modulus, obtained from the stress–strain gradient at low strains.

d Ultimate tensile toughness, determined as the area under the stress–strain curves.

Tensile testing showed that blend samples containing 10 wt % **PCPC-9** showed a decrease in both tensile strength and strain compared to **PCPC-76**. As a consequence, the ultimate tensile toughness (*U*_t_) of the 10 wt % blend was approximately half that of neat **PCPC-76** and fell to as low as 30% of the original when equal amounts of **PCPC-9** and **PCPC-76** were blended together. Overall, the addition of low molecular weight, unentangled PCPC is detrimental to the mechanical properties. **PCPC-16** blends returned slightly different results: blending 10 wt % of this material with **PCPC-76** did not significantly affect tensile properties; higher concentrations, however, caused a similar but less severe decrease in performance compared to **PCPC-16** (Figure 3c,d). Both cases show that it is best to minimize the presence of low molecular weight chains to achieve strong, tough PCPC. These experiments suggest that the limit is below 30% for low molecular weight, entangled **PCPC-16,** although the limiting quantity would likely vary depending on the relative molecular weights in the mixture. It seems most important to minimize processes that might result in the formation of short, unentangled chain content, as it led to an immediate drop in the performance of the high molecular weight PCPC.

**Figure 3:**
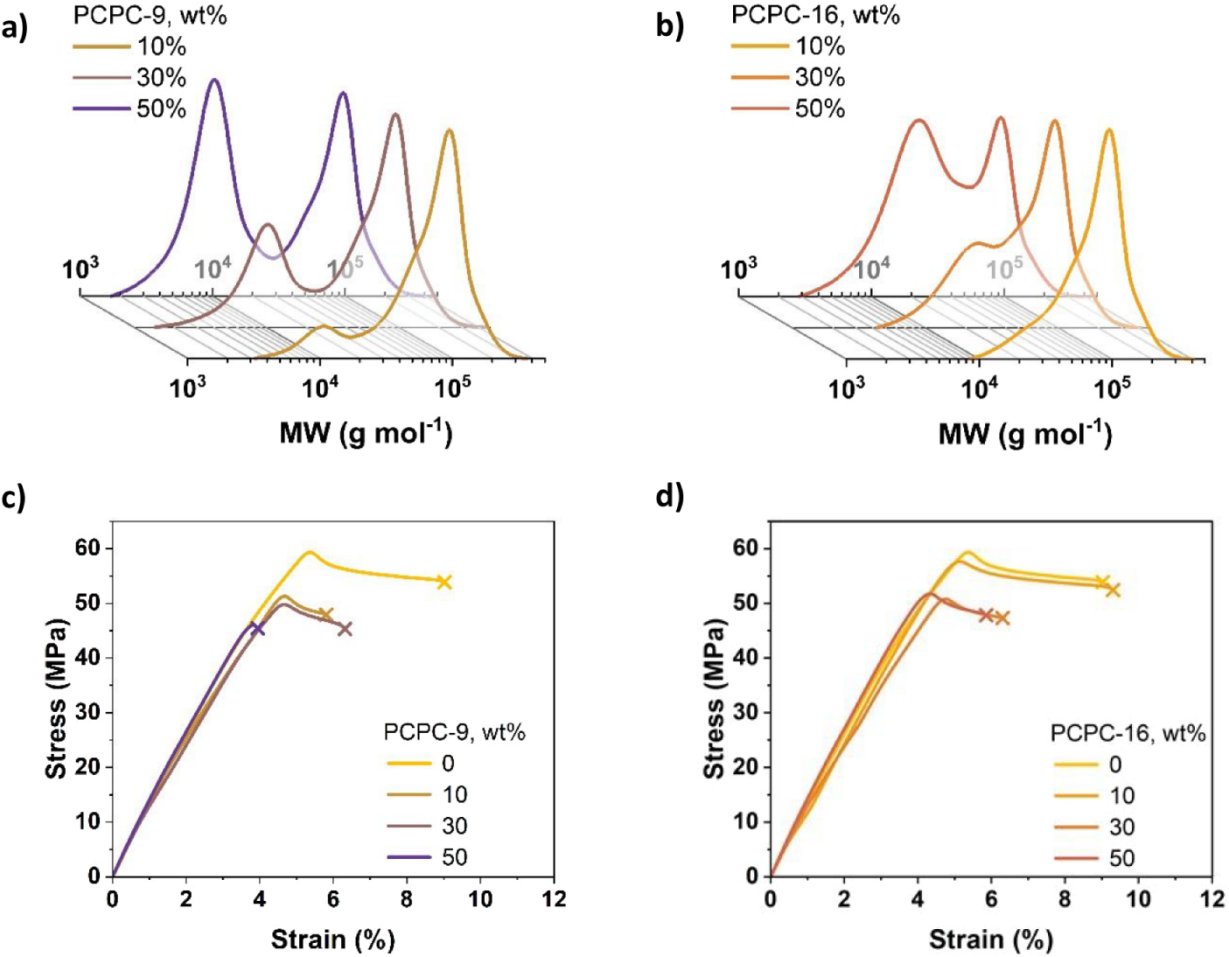
GPC and tensile data for wide-gap bimodal PCPC samples. (a) GPC data of **PCPC-9** blends, (b) GPC data of **PCPC-16** blends, both with **PCPC-76**. (c) Representative stress–strain curves of **PCPC-9** blends with **PCPC-76**. (d) Representative stress–strain curves of **PCPC-16** blends with **PCPC-76**. Tensile testing was carried out at a 10 mm min^–1^ crosshead velocity in both cases.

Chemical recyclability of PCPC back to its monomers, CPO and CO_2_, has been demonstrated using various catalyst systems, including the LCo­(II)­Mg­(II)­(OAc)_2_ polymerization catalyst used in this work (Scheme 2).^10,13^ To investigate any influence of PCPC MWD on recycling rates, narrow-gap bimodal PCPC samples were combined with the LCo­(II)­Mg­(II)­(OAc)_2_ catalyst at a 1:300 catalyst-to-polycarbonate repeat unit ratio (1.9 wt % catalyst), and the resulting films were dried thoroughly. PCPC depolymerization was conducted at 140 °C and investigated using TGA-FTIR spectroscopy, as described in prior catalytic chemical recycling papers.^10^ In every case, the PCPC underwent 80% mass loss in under 9 min, and complete polycarbonate mass loss was achieved between 10 and 15 min (Figure 4). Mass loss versus time data were fit with exponential models to obtain *k*_obs_ values from 12 to 18 h^–1^, corresponding to high turnover frequencies (TOF) from 1600 to 2400 h^–1^ (Table S4). There were no particular trends between rates and PCPC molecular weight distribution (modality), with minor rate differences likely a consequence of experimental errors for fast reactions at small scale. The decomposition products were confirmed as cyclopentene oxide and carbon dioxide by IR spectroscopy, validated by independent calibration using pure samples of those compounds (Figure S44). Previous studies established that, at lab scale, CPO can be isolated and repolymerized to support the future potential for closed-loop PCPC recycling-production.^13,42^ Here, the key finding is that, regardless of the molecular weight distribution, the PCPC samples are always efficiently and selectively recycled to the monomers.

**Scheme 2:**
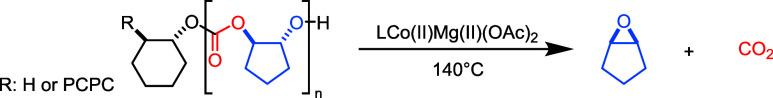
Chemical Recycling of the PCPC to Yield CPO and CO_2_ [Fn sch1-fn2]

a Conditions: [cat]:[PCPC repeat unit] 1:300 at 140 °C, monitored by TGA-IR instrumentation (see Supporting Information for details)

**Figure 4:**
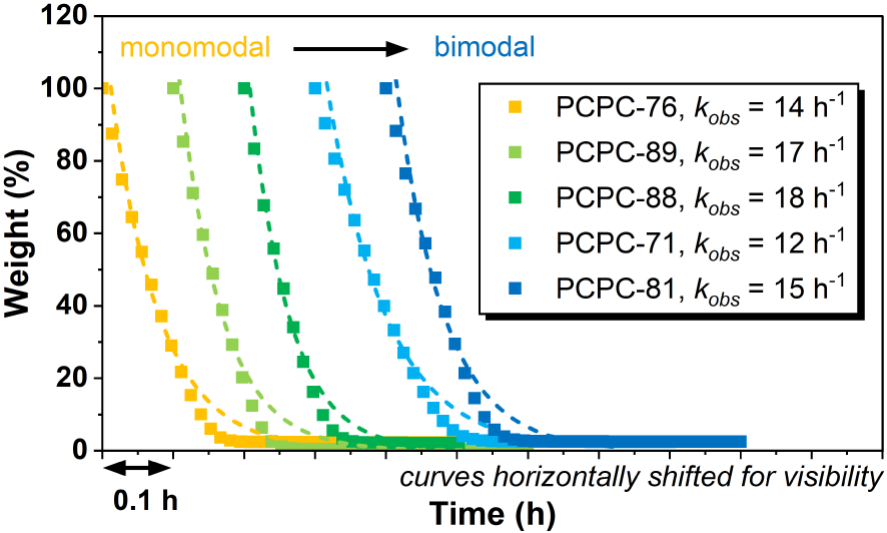
Chemical recycling of the narrow-gap bimodal MWD PCPC series. Conditions: 1:300 [catalyst]:[PCPC repeat unit] loading (1.9 wt % catalyst), 140 °C. Data series were shifted along the *X*-axis for visibility. *k*_obs_ values were obtained by fitting data between 20 and 80% weight loss as an exponential decay.

## Conclusions

Poly­(cyclopentene carbonate) was efficiently and selectively prepared from cyclopentene oxide/CO_2_ ring-opening copolymerization, using an LCo­(II)­Mg­(II)­(OAc)_2_ catalyst operating with both high turnover numbers (TON = 18,000) and frequencies (TOF = 120–460 h^–1^). A series of PCPC samples were produced with molecular weights from 9 to 80 kg mol^–1^ and monomodal distributions, provided that the relative catalyst:diol ratios were 1:10 or higher. A series of PCPC samples showing high molecular weights (*M*_*n*_ ∼ 81 kg mol^–1^) and narrow-gap bimodality were prepared using a catalyst:(monoalcohol + diol) ratio of 1:8, with systematically varied alcohol and diol amounts controlling the relative intensities of the distributions. These samples all showed high tensile strength, stiffness, and glass transition temperature values, regardless of the relative intensities of the narrow-gap molecular weight distributions. A second series of PCPC samples modeling wide-gap bimodality were prepared by blending known amounts of lower molecular weight PCPC (*M*_*n*_ = 9 or 16 kg mol^–1^) with high molecular weight PCPC (*M*_*n*_ = 76 kg mol^–1^). These samples showed compromised tensile strengths and glass transition temperatures if the additional low-molecular-weight chains are below the entanglement molecular weight. High-molecular-weight PCPC can tolerate some (30 wt %) shorter but entangled chains, with no significant change to either *T*_g_ or tensile properties. Chemical recycling of high-molecular-weight PCPC back to CPO and CO_2_ was fast and selective, regardless of the polymer molecular weight distribution. Overall, the LCo­(II)­Mg­(II)­(OAc)_2_ catalyst is fast, selective, and tolerant for both polymerization and depolymerization of PCPC. In terms of material properties, PCPC can tolerate bimodal molecular weight distributions, provided the constituent chains are entangled. These results may be significant, since the molecular weight distribution and overall molecular weight depend upon the presence/absence of reagent contaminants, including water. Therefore, establishing catalysts that operate efficiently in the presence of these impurities, and producing materials that maintain good properties over a range of molecular weight distributions, are important for the future development of effective manufacturing processes.

## Supplementary Material



## Notes

Materials, methods, and experimental details. Supplementary tables and figures, including gel-permeation chromatography, thermal-mechanical characterization, and depolymerization data (DOCX)
