# Supplementary material for: Effects of Molecular Weight Distribution on the Thermal–Mechanical Performance and Recycling of CO2‑Derived Poly(cyclopentene carbonate)
Source: Macromolecules. 2026 Jan 12;59(2):793–801. doi: 10.1021/acs.macromol.5c03136 (PMC12854764; doi:10.1021/acs.macromol.5c03136)
Supplement: Supplementary file 1 [file ma5c03136_si_001.docx]

Supporting Information

**Effects of molecular weight distribution on thermal-mechanical performance and recycling of CO_2_-derived poly(cyclopentene carbonate)**

Balázs Striker,^[a]^ Alexander R. Craze,^[a]^ Kam C. Poon,^[a]^ Thomas M. McGuire,^[a]^ Kristian L. Mears,^[a]^ and Charlotte K. Williams*^[a]^

^[a]^Department of Chemistry, University of Oxford, Oxford OX1 3TA, United Kingdom

Corresponding author email address: charlotte.williams@chem.ox.ac.uk

# **Contents**

[Contents	2](#_Toc216376989)

[Materials	4](#_Toc216376990)

[Methods	4](#_Toc216376991)

[Experimental details	5](#_Toc216376992)

[**Synthesis of LCo(II)Mg(II)(OAc)_2_ catalyst**	5](#_Toc216376993)

[**General procedure for PCPC synthesis**	6](#_Toc216376994)

[**Table S1**. Synthesis of monomodal PCPCs	6](#_Toc216376995)

[**Table S2**. Synthesis of narrow-gap bimodal PCPCs.	6](#_Toc216376996)

[**Protocol for PCPC blending**	6](#_Toc216376997)

[**General procedure for depolymerization of narrow-gap bimodal PCPC**	7](#_Toc216376998)

[Equations	8](#_Toc216376999)

[**Equation S1.** Calculation of standard error.	8](#_Toc216377000)

[**Equation S2.** Flory-Fox equation describing the relationship between *T*_g_ and *M*_n_.	8](#_Toc216377001)

[**Equation S3.** Fox equation describing the relationship between *T*_g_ and the weight fraction of binary blends of miscible polymers.	8](#_Toc216377002)

[Supplementary Figures and Tables	9](#_Toc216377003)

[**Scheme S1.** Chain initiation and propagation in CO_2_/CPO ROCOP using cobalt-magnesium diacetate catalyst LCo(II)Mg(II)(OAc)_2_.	9](#_Toc216377004)

[**Figure S2**. GPC data of **PCPC-9**, synthesised with 1:50:10 000 [cat]:[CHD]:[CPO] loading.	10](#_Toc216377005)

[**Figure S3**. GPC data of **PCPC-16**, synthesised with 1:50:10 000 [cat]:[CHD]:[CPO] loading.	10](#_Toc216377006)

[**Figure S4**. GPC data of **PCPC-37**, synthesised with 1:23:10 000 [cat]:[CHD]:[CPO] loading.	10](#_Toc216377007)

[**Figure S5**. GPC data of **PCPC-76**, synthesised with 1:0:8:20 000 [cat]:[CHM]:[CHD]:[CPO] loading.	11](#_Toc216377008)

[**Figure S6**. GPC data of **PCPC-89**, synthesised with 1:2:6:20 000 [cat]:[CHM]:[CHD]:[CPO] loading.	11](#_Toc216377009)

[**Figure S7**. GPC data of **PCPC-88**, synthesised with 1:4:4:20 000 [cat]:[CHM]:[CHD]:[CPO] loading.	11](#_Toc216377010)

[**Figure S8**. GPC data of **PCPC-71**, synthesised with 1:6:2:20 000 [cat]:[CHM]:[CHD]:[CPO] loading.	12](#_Toc216377011)

[**Figure S9**. GPC data of **PCPC-81**, synthesised with 1:8:0:20 000 [cat]:[CHM]:[CHD]:[CPO] loading.	12](#_Toc216377012)

[**Figure S10**. GPC data of 10 wt% **PCPC-9** in **PCPC-76**.	13](#_Toc216377013)

[**Figure S11**. GPC data of 30 wt% **PCPC-9** in **PCPC-76**.	13](#_Toc216377014)

[**Figure S12**. GPC data of 50 wt% **PCPC-9** in **PCPC-76**.	13](#_Toc216377015)

[**Figure S13**. GPC data of 10 wt% **PCPC-16** in **PCPC-76**.	14](#_Toc216377016)

[**Figure S14**. GPC data of 30 wt% **PCPC-16** in **PCPC-76**.	14](#_Toc216377017)

[**Figure S15**. GPC data of 50 wt% **PCPC-16** in **PCPC-76**.	14](#_Toc216377018)

[**Figure S16**. DSC data of monomodal PCPC series.	15](#_Toc216377019)

[**Figure S17**. TGA data of monomodal PCPC series.	15](#_Toc216377020)

[**Figure S18**. DSC data of narrow-gap bimodal PCPC series.	16](#_Toc216377021)

[**Figure S19**. TGA data of narrow-gap bimodal PCPC series.	16](#_Toc216377022)

[**Figure S20**. DSC data of **PCPC-9** blends in **PCPC-76**.	17](#_Toc216377023)

[**Figure S21**. DSC data of **PCPC-16** blends in **PCPC-76**.	17](#_Toc216377024)

[**Figure S22**. Wide-gap PCPC blend *T*_g_-s compared to the Fox equation applicable for miscible binary polymer blends.	18](#_Toc216377025)

[**Figure S23**. Complete tensile data for **PCPC-76**.	19](#_Toc216377026)

[**Figure S24**. Complete tensile data for **PCPC-89**.	19](#_Toc216377027)

[**Figure S25**. Complete tensile data for **PCPC-88**.	20](#_Toc216377028)

[**Figure S26**. Complete tensile data for **PCPC-71**.	20](#_Toc216377029)

[**Figure S27**. Complete tensile data for **PCPC-81**.	21](#_Toc216377030)

[**Table S3**. Tensile properties and *T*_g_-s of narrow-gap bimodal PCPCs, compared to previous reports of PCPC thermal-mechanical properties.	21](#_Toc216377031)

[**Figure S28**. Complete tensile data for **PCPC-9** blends in **PCPC-76**.	22](#_Toc216377032)

[**Figure S29**. Complete tensile data for **PCPC-16** blends in **PCPC-76**.	23](#_Toc216377033)

[**Figure S36**. Oscillatory temperature ramp of **PCPC-76** (0.1% strain, 1.0 Hz, 3 °C min^−1^). Top: dynamic moduli, G’ and G’’. Bottom: tan(δ).	27](#_Toc216377034)

[**Figure S37**. Oscillatory temperature ramp of **PCPC-81** (0.1% strain, 1.0 Hz, 3 °C min^−1^). Top: dynamic moduli, G’ and G’’. Bottom: tan(δ).	28](#_Toc216377035)

[**Figure S41**. Complex viscosity of **PCPC-76** and **PCPC-81**, obtained from oscillatory temperature ramp experiments.	28](#_Toc216377036)

[**Figure S39**. Solid-state depolymerization of **PCPC-76** using LCo(II)Mg(II)(OAc)_2_ catalyst at 1:300 [catalyst]:[PCPC repeat unit] loading at 140 ˚C. The data between 80-20% mass loss is fit to exponentials to determine *k*_obs_.	29](#_Toc216377037)

[**Figure S41**. Solid-state depolymerization of **PCPC-88** using LCo(II)Mg(II)(OAc)_2_ catalyst at 1:300 [catalyst]:[PCPC repeat unit] loading at 140 ˚C. The data between 80-20% mass loss is fit to exponentials to determine *k*_obs_.	30](#_Toc216377038)

[**Figure S43**. Solid-state depolymerization of **PCPC-81** using LCo(II)Mg(II)(OAc)_2_ catalyst at 1:300 [catalyst]:[PCPC repeat unit] loading at 140 ˚C. The data between 80-20% mass loss is fit to exponentials to determine *k*_obs_.	31](#_Toc216377039)

[**Table S4**. Depolymerization rates of narrow-gap bimodal PCPCs.	31](#_Toc216377040)

[**Figure S44**. Representative vapour-phase TGA-FTIR analysis of solid-state PCPC depolymerization compared against FTIR spectra of CPO and CO_2_ monomer standards.	32](#_Toc216377041)

[References	32](#_Toc216377042)

# Materials

Solvents used in synthesis under inert conditions were collected from a solvent purification system (SPS), degassed by three freeze-pump-thaw cycles, and stored under 3 Å molecular sieves, under a N_2_ atmosphere. H_2_L ligand was synthesised according to a literature procedure.^1^ Cyclopentene oxide (CPO) was dried over CaH_2_ for 1 day then distilled under argon. The CPO was then distilled again with the addition of ^n^BuLi in hexanes then distilled one final time under argon. This process was then repeated again to provide high-purity CPO. 1,2-Cyclohexanediol (CHD) was recrystallized from ethyl acetate. Cyclohexanol (CHM) was dried over 3 Å activated molecular sieves and degassed by three freeze-pump-thaw cycles, and stored under an Ar atmosphere. All other reagents were used as received. CO_2_ gas (BOC, CP grade, 99.995 %) was passed through two carbon dioxide purifiers (2 µ internal frit, P600 produced by VICI metronics) at point of use. For polymerization reactions, 25 and 100 mL high pressure Parr reactors were used, equipped with pressure gauge and overhead stirring. The reactor vessels have been pre-dried at 140 °C and the reactants were loaded into the reactor under inert conditions. The reactors were cycled onto a triple manifold CO_2_/N_2_/vacuum high pressure gas line for high pressure CO_2_ polymerization reactions.

# Methods

“Inert conditions” describe working in a water-free (<0.1 ppm) and oxygen-free (<10.0 ppm) atmosphere, either in an MBraun glovebox filled with nitrogen or a dual manifold Ar-vacuum Schlenk line. All manipulations, unless otherwise stated, were carried out in standard borosilicate glass equipment, pre-dried at 140 °C when inert conditions were used.

**NMR Spectroscopy**: ^1^H NMR spectra were obtained using Bruker Ascend 400 NMR spectrometers. All spectra were recorded at 298 K.

**Gel Permeation Chromatography (GPC)**: GPC analysis was carried out on a Shimadzu LC-20AD instrument, equipped with a Refractive Index (RI) detector and two PSS SDV 5 μm linear M columns, with an eluent of inhibitor-free THF, at 30 °C and at a flow rate of 1.0 mL min-1. Monodisperse polystyrene standards were used for calibration.

**Differential Scanning Calorimetry (DSC):** DSC measurements were made using a DSC 25 (TA Instruments). Samples were heated from -80 °C to 200 °C, at a rate of 10 °C min^-1^ under N_2_ flow (80 mL min^-1^) followed by a 5-minute isotherm at 200 °C to erase thermal history. Samples were subsequently cooled to -80 °C, at a rate of 10 °C min^-1^ and kept at -80 °C for a further 5 minutes, followed by a heating-cooling procedure from -80 °C to 200 °C, at a rate of 10 °C min^-1^. Glass transition temperatures (*T*_g_) are reported as the midpoint of the transition taken from the second heating cycle.

**Thermogravimetric Analysis (TGA):** TGA measurements were made on a Discovery TGA5500 (TA Instruments), heating at 10 ˚C min^-1^ from 35 to 600 ˚C under N_2_ flow (25 mL min^-1^). The polymer samples were purified and vacuum dried at 110 °C prior to TGA measurements.

**TGA-FTIR:** Measurements were collected on a Nicolet iS20 FTIR spectrometer (Thermo Scientific Instruments) coupled to a TGA5500 (TA instruments). The mass flow of the TGA was set to 30 mL min^‑1^. Spectra were recorded, between 400–4000 cm^–1^, with 10 scans per spectrum, at a resolution of 8 cm^‑1^.

**Compression moulding:** Polymer films were processed using a Carver mini CH CE Press with heated plates. The purified, dry polymers/polymer blends were heated to 130 °C and compressed at 1 ton pressure for 5 minutes. This was carried out three subsequent times, turning the heating off after 5 minutes after the final pressing and leaving the sample slowly cool to room temperature under pressure (for a minimum of 5 hours) to obtain polycarbonate sheets of 0.2-0.5 mm thickness.

**Uniaxial Tensile Testing:** Tests were carried out using an Instron 8600 series universal testing system, using a 50 N load cell and 250 N pneumatic grips. ISO 527-2 type 5B specimens (“dog bone”-shaped) were cut from compression moulded sheets using a Zwick ZCP020 cutting press, equipped with a cutting device confirming to the standard. Uniaxial extension experiments (10 mm min^-1^ cross-head velocity) were run according to ISO 527. A minimum of 5 specimens from each material sample were tested and the mean values of maximum tensile stress, elongation at break, Young’s modulus and ultimate tensile toughness were obtained.

**Frequency Sweep Measurements and Time-Temperature superposition:** The measurements were performed on an ARES-G2 rheometer (TA Instruments) between two stainless steel 8 mm parallel plates. Where ductility permitted, clear round disks 8 mm in diameter were punch-cut from compression moulded sheets of polycarbonate. In case of **PCPC-9** which was highly brittle, solid samples were deposited between the plates and melted to form an even layer. Consecutive oscillatory frequency sweep measurements were performed from 100 °C to 200 °C with 10 °C intervals at 0.1% strain, 0.01-100 Hz with 1 min equilibration time between runs. The datasets were then combined into the corresponding time-temperature superposition (TTS) master curves using the software TRIOS, version 5.7.1.74 (TA Instruments) and referenced at 140 °C. The shift factors (*a*_T_) obtained, and used for master curve construction, were fitted to the Williams-Landel-Ferry equation:

$$\log a_{T}= \frac{-C_{1}(T-T_{ref})}{C_{2}+T-T_{ref}}$$

Where *T*_ref_ is the reference temperature (140 °C) used for master curve construction.

**Oscillatory Temperature Ramp Measurements:** The measurements were performed on an ARES-G2 rheometer (TA Instruments) between two stainless steel 8 mm parallel plates. Specimens were prepared similarly to Frequency Sweeps. The samples were heated and equilibrated at 200 °C. The samples were then cooled at a 3 °C min^−1^ rate and oscillated at 0.1% strain, 1.0 Hz frequency.

# Experimental details

## **Synthesis of LCo(II)Mg(II)(OAc)_2_ catalyst**

To a solution of H_2_L (1.43 g, 2.59 mmol) in dry 2-methyltetrahydrofuran (20 mL) was added Mg(HMDS)_2_(THF)_2_ (1.27 g, 2.59 mmol) and the mixture was stirred under inert conditions. After 2 hours and 40 minutes, Co(OAc)_2_ (0.459 g, 2.59 mmol) was added and the mixture was heated to 100 °C. After 19 hours the reaction was taken off the heat and the solvent was evaporated under vacuum. The product was then washed with dry pentane three times and dried under vacuum to give LCo(II)Mg(II)(OAc)_2_ (1.77 g, 91%, 2.35 mmol).

## **General procedure for PCPC synthesis**

Under inert conditions, LCo(II)Mg(II)(OAc)_2_ and varying ratios of cyclohexanol and 1,2-cyclohexanediol were dissolved in a mixture of CPO (9.30 mL, 106 mmol) and dry toluene (8.4 mL) and the mixture was then loaded into a high-pressure reactor, pressurised to 20 bar with CO_2_ and heated to 100 °C under stirring. The reaction was periodically refilled with CO_2_ to 20 bar pressure. After 39-68 hours, the reaction was cooled to room temperature and depressurised. The crude mixture was precipitated in MeOH (300 mL), and subsequently re-precipitated from THF/MeOH. The product was then redissolved in THF (400 mL) and filtered through two short columns of silica. The filtrate was then concentrated, solvent cast and air dried overnight then dried at 110 °C under vacuum for 16 hours to yield poly(cyclopentene carbonate) as a white solid with the exception of **PCPC-9** that was brown and **PCPC-16** that was off-white.

## **Table S1**. Synthesis of monomodal PCPCs

| **Polymer** | **[cat]:[CHD]:[CPO]** | **Catalyst**  **(mg, *µmol*)** | **CHD**  **(mg, *µmol*)** | **R. time**  **(h)** | **Conv.^[a]^** | **Yield**  **(g, [%])** | **TOF^[b]^**  **(h^-1^)** |
| --- | --- | --- | --- | --- | --- | --- | --- |
| **PCPC-76** | 1 : 8 : 20 000 | 4.1, *5.4* | 5.0, *43* | 39 | 90% | 10.58 [78%] | 460 |
| **PCPC-37** | 1 : 23 : 10 000 | 4.8, *6.3* | 16.8, *145* | 42 | 94% | 6.3 [78%] | 230 |
| **PCPC-16** | 1 : 50 : 10 000 | 4.7, *6.3* | 35.2, *303* | 68 | 89% | 4 [50%] | 130 |
| **PCPC-9** | 1 : 50 : 10 000 | 4.8, *6.3* | 36.7, *315* | 48 | 57% | 3.01 [37%] | 120 |

^[a]^([PCPC] + [cyclic carbonate])/([CPO] + [PCPC] + [cyclic carbonate]), determined by ^1^H NMR spectroscopy. ^[b]^TON/reaction time = (conversion×[CPO]_i_/[catalyst])/reaction time, determined by ^1^H NMR spectroscopy.

## **Table S2**. Synthesis of narrow-gap bimodal PCPCs.

| **Polymer** | **[cat]:[CHM]:[CHD]:[CPO]** | **Catalyst**  **(mg, *µmol*)** | **CHM**  **(µL, *µmol*)** | **CHD**  **(mg, *µmol*)** | **R. time (h)** | **Conv.^[a]^** | **Yield**  **(g, [%])** |
| --- | --- | --- | --- | --- | --- | --- | --- |
| **PCPC-76** | 1 : 0 : 8 : 20 000 | 4.1, *5.4* | 0 | 5.0, *43* | 39 | 90% | 10.58 [78%] |
| **PCPC-89** | 1 : 2 : 6 : 20 000 | 4.0, *5.3* | 1.1, *11* | 3.7, *32* | 44 | 92% | 10.42 [77%] |
| **PCPC-88** | 1 : 4 : 4 : 20 000 | 4.0, *5.3* | 2.2, *21* | 2.5, *21* | 44 | 85% | 9.96 [73%] |
| **PCPC-71** | 1 : 6 : 2 : 20 000 | 4.0, *5.3* | 3.3, *32* | 1.2, *11* | 44 | 89% | 10.3 [76%] |
| **PCPC-81** | 1 : 8 : 0 : 20 000 | 4.1, *5.4* | 4.4, *43* | 0 | 45 | 88% | 7.96 [57%] |

^[a]^([PCPC] + [cyclic carbonate])/([CPO] + [PCPC] + [cyclic carbonate]), determined by ^1^H NMR spectroscopy.

## **Protocol for PCPC blending**

Blends of **PCPC-9** or **PCPC-16** and **PCPC-76** were prepared using stock solutions of the polymers in THF. The solution mixtures were concentrated, solvent cast, air-dried at 70 °C then vacuum dried at 120 °C.

## **General procedure for depolymerization of narrow-gap bimodal PCPC**

To 20 mg PCPC, a stock solution of LCo(II)Mg(II)(OAc)_2_ (0.20 mL of 1.98 mg mL^-1^ in THF, 1 eq per 300 PCPC unit) was added under inert conditions, then THF was added until the sample was fully dissolved. The solvent was fully evaporated under vacuum. The resulting film was transferred into a 100 µL platinum TGA pan and heated to 140 °C. Conversion was monitored by TGA-FTIR.

# Equations

## **Equation S1.** Calculation of standard error.

$$\sigma_{\bar{x}}= \frac{\sigma}{\sqrt{n}}$$

Where $\sigma_{\bar{x}}$ is the standard error, $\sigma$ is the population standard deviation and $n$ is the number of samples (repeats).

## **Equation S2.** Flory-Fox equation describing the relationship between *T*_g_ and *M*_n_.

$$T_{g}= T_{g,\infty}-\frac{K}{M_{n}}$$

Where *T*_g_ is the glass transition temperature, *T*_g,∞_ is the maximum glass transition temperature at theoretical infinite chai lengths, *M*_n_ is the umber average molecular weight and *K* is the empirical free volume parameter.^2^

## **Equation S3.** Fox equation describing the relationship between *T*_g_ and the weight fraction of binary blends of miscible polymers.

$$\frac{1}{T_{g}}= \frac{x_{1}}{T_{g,1}}+\frac{x_{2}}{T_{g,2}}$$

Where *T*_g_ is the glass transition temperature of the blend, *T*_g,1_ and *T*_g,1_ are the individual glass transition temperatures of the constituent polymers and *x*_1_ and *x*_2_ are the weight fractions of the respective constituent polymers (*x*_1_ + *x*_2_ = 1).^3^

# Supplementary Figures and Tables

## **Scheme S1.** Chain initiation and propagation in CO_2_/CPO ROCOP using cobalt-magnesium diacetate catalyst LCo(II)Mg(II)(OAc)_2_.

**Figure S1.** Representative ^1^H NMR spectrum of purified poly(cyclopentene carbonate).

c, b’

b

c

b, b’

a

a

|  |  |  |  | |
| --- | --- | --- | --- | --- |
|  | ***M*_n_**  **(kg mol^-1^)** | ***M*_w_**  **(kg mol^-1^)** | ***Đ*** |  |
|  |  |  |  | |
|  | 9.3 | 11.2 | 1.21 | |
|  |  |  |  | |
|  |  |  |  | |
|  |  |  |  | |
|  |  |  |  | |
|  |  |  |  | |
|  |  |  |  | |
|  |  |  |  | |
|  |  |  |  | |
|  |  |  |  | |

## **Figure S2**. GPC data of **PCPC-9**, synthesised with 1:50:10 000 [cat]:[CHD]:[CPO] loading.

|  |  |  |  | |
| --- | --- | --- | --- | --- |
|  | ***M*_n_**  **(kg mol^-1^)** | ***M*_w_**  **(kg mol^-1^)** | ***Đ*** |  |
|  |  |  |  | |
|  | 16.4 | 20.8 | 1.27 | |
|  |  |  |  | |
|  |  |  |  | |
|  |  |  |  | |
|  |  |  |  | |
|  |  |  |  | |
|  |  |  |  | |
|  |  |  |  | |
|  |  |  |  | |
|  |  |  |  | |

## **Figure S3**. GPC data of **PCPC-16**, synthesised with 1:50:10 000 [cat]:[CHD]:[CPO] loading.

|  |  |  |  | |
| --- | --- | --- | --- | --- |
|  | ***M*_n_**  **(kg mol^-1^)** | ***M*_w_**  **(kg mol^-1^)** | ***Đ*** |  |
|  |  |  |  | |
|  | 36.8 | 45.5 | 1.24 | |
|  |  |  |  | |
|  |  |  |  | |
|  |  |  |  | |
|  |  |  |  | |
|  |  |  |  | |
|  |  |  |  | |
|  |  |  |  | |
|  |  |  |  | |
|  |  |  |  | |

## **Figure S4**. GPC data of **PCPC-37**, synthesised with 1:23:10 000 [cat]:[CHD]:[CPO] loading. Note that the minor higher molecular weight shoulder may result from viscosity limitations (stirring issues).

|  |  | |  |  |  | |
| --- | --- | --- | --- | --- | --- | --- |
|  | **stage** | | ***M*_n_**  **(kg mol^-1^)** | ***M*_w_**  **(kg mol^-1^)** | ***Đ*** |  |
|  | **crude** |  |  |  |  | |
|  |  |  | 75.7 | 93.7 | 1.24 | |
|  |  |  |  |  |  | |
|  |  |  |  |  |  | |
|  | **purified** |  | 73.1 | 91.6 | 1.25 | |
|  |  |  |  |  |  | |
|  |  |  |  |  |  | |
|  | **processed** |  | 70.5 | 88.4 | 1.25 | |
|  |  |  |  |  |  | |
|  |  |  |  |  |  | |
|  |  |  |  |  |  | |

## **Figure S5**. GPC data of **PCPC-76**, synthesised with 1:0:8:20 000 [cat]:[CHM]:[CHD]:[CPO] loading. Note that the minor higher molecular weight shoulder may result from viscosity limitations (stirring issues).

|  |  | |  |  |  | |
| --- | --- | --- | --- | --- | --- | --- |
|  | **stage** | | ***M*_n_**  **(kg mol^-1^)** | ***M*_w_**  **(kg mol^-1^)** | ***Đ*** |  |
|  | **crude** |  |  |  |  | |
|  |  |  | 89.3 | 106 | 1.19 | |
|  |  |  |  |  |  | |
|  |  |  |  |  |  | |
|  | **purified** |  | 80.0 | 99.9 | 1.25 | |
|  |  |  |  |  |  | |
|  |  |  |  |  |  | |
|  | **processed** |  | 76.1 | 97.9 | 1.29 | |
|  |  |  |  |  |  | |
|  |  |  |  |  |  | |
|  |  |  |  |  |  | |

## **Figure S6**. GPC data of **PCPC-89**, synthesised with 1:2:6:20 000 [cat]:[CHM]:[CHD]:[CPO] loading. Note that the minor higher molecular weight shoulder may result from viscosity limitations (stirring issues).

|  |  | |  |  |  | |
| --- | --- | --- | --- | --- | --- | --- |
|  | **stage** | | ***M*_n_**  **(kg mol^-1^)** | ***M*_w_**  **(kg mol^-1^)** | ***Đ*** |  |
|  | **crude** | peak 1 | 51.2 | 54.5 | 1.06 | |
|  |  | peak 2 | 115 | 125 | 1.09 | |
|  |  | total | 88.1 | 108 | 1.23 | |
|  |  | peak 1 | 48.3 | 54.0 | 1.12 | |
|  | **purified** | peak 2 | 117 | 128 | 1.09 | |
|  |  | total | 85.2 | 108 | 1.27 | |
|  |  | peak 1 | 52.3 | 55.5 | 1.05 | |
|  | **processed** | peak 2 | 116 | 128 | 1.10 | |
|  |  | total | 91.6 | 111 | 1.22 | |
|  |  |  |  |  |  | |
|  |  |  |  |  |  | |

## **Figure S7**. GPC data of **PCPC-88**, synthesised with 1:4:4:20 000 [cat]:[CHM]:[CHD]:[CPO] loading. Note that the minor higher molecular weight shoulder may result from viscosity limitations (stirring issues).

|  |  | |  |  |  | |
| --- | --- | --- | --- | --- | --- | --- |
|  | **stage** | | ***M*_n_**  **(kg mol^-1^)** | ***M*_w_**  **(kg mol^-1^)** | ***Đ*** |  |
|  | **crude** | peak 1 | 40.0 | 50.0 | 1.25 | |
|  |  | peak 2 | 111 | 121 | 1.09 | |
|  |  | total | 70.5 | 98.1 | 1.39 | |
|  |  | peak 1 | 44.8 | 52.3 | 1.18 | |
|  | **purified** | peak 2 | 113 | 121 | 1.07 | |
|  |  | total | 73.7 | 97.1 | 1.32 | |
|  |  | peak 1 | 46.2 | 50.6 | 1.10 | |
|  | **processed** | peak 2 | 107 | 115 | 10.8 | |
|  |  | total | 73.5 | 92.9 | 1.26 | |
|  |  |  |  |  |  | |
|  |  |  |  |  |  | |

## **Figure S8**. GPC data of **PCPC-71**, synthesised with 1:6:2:20 000 [cat]:[CHM]:[CHD]:[CPO] loading. Note that the minor higher molecular weight shoulder may result from viscosity limitations (stirring issues).

|  |  | |  |  |  | |
| --- | --- | --- | --- | --- | --- | --- |
|  | **stage** | | ***M*_n_**  **(kg mol^-1^)** | ***M*_w_**  **(kg mol^-1^)** | ***Đ*** |  |
|  | **crude** | peak 1 | 52.0 | 56.9 | 1.09 | |
|  |  | peak 2 | 120 | 132 | 1.09 | |
|  |  | total | 80.8 | 104 | 1.28 | |
|  |  | peak 1 | 52.9 | 58.5 | 1.10 | |
|  | **purified** | peak 2 | 123 | 132 | 1.07 | |
|  |  | total | 79.4 | 101 | 1.28 | |
|  |  | peak 1 | 50.8 | 56.9 | 1.13 | |
|  | **processed** | peak 2 | 122 | 132 | 1.08 | |
|  |  | total | 76.8 | 101 | 1.31 | |
|  |  |  |  |  |  | |
|  |  |  |  |  |  | |

## **Figure S9**. GPC data of **PCPC-81**, synthesised with 1:8:0:20 000 [cat]:[CHM]:[CHD]:[CPO] loading. Note that the minor higher molecular weight shoulder may result from viscosity limitations (stirring issues).

|  |  |  |  | |
| --- | --- | --- | --- | --- |
|  | ***M*_n_**  **(kg mol^-1^)** | ***M*_w_**  **(kg mol^-1^)** | ***Đ*** |  |
|  |  |  |  | |
|  | 39.2 | 76.5 | 1.95 | |
|  |  |  |  | |
|  |  |  |  | |
|  |  |  |  | |
|  |  |  |  | |
|  |  |  |  | |
|  |  |  |  | |
|  |  |  |  | |
|  |  |  |  | |
|  |  |  |  | |

## **Figure S10**. GPC data of 10 wt% **PCPC-9** in **PCPC-76**.

|  |  |  |  | |
| --- | --- | --- | --- | --- |
|  | ***M*_n_**  **(kg mol^-1^)** | ***M*_w_**  **(kg mol^-1^)** | ***Đ*** |  |
|  |  |  |  | |
|  | 20.1 | 61.3 | 3.05 | |
|  |  |  |  | |
|  |  |  |  | |
|  |  |  |  | |
|  |  |  |  | |
|  |  |  |  | |
|  |  |  |  | |
|  |  |  |  | |
|  |  |  |  | |
|  |  |  |  | |

## **Figure S11**. GPC data of 30 wt% **PCPC-9** in **PCPC-76**.

|  |  |  |  | |
| --- | --- | --- | --- | --- |
|  | ***M*_n_**  **(kg mol^-1^)** | ***M*_w_**  **(kg mol^-1^)** | ***Đ*** |  |
|  |  |  |  | |
|  | 14.7 | 48.1 | 3.28 | |
|  |  |  |  | |
|  |  |  |  | |
|  |  |  |  | |
|  |  |  |  | |
|  |  |  |  | |
|  |  |  |  | |
|  |  |  |  | |
|  |  |  |  | |
|  |  |  |  | |

## **Figure S12**. GPC data of 50 wt% **PCPC-9** in **PCPC-76**.

|  |  |  |  | |
| --- | --- | --- | --- | --- |
|  | ***M*_n_**  **(kg mol^-1^)** | ***M*_w_**  **(kg mol^-1^)** | ***Đ*** |  |
|  |  |  |  | |
|  | 58.2 | 83.4 | 1.43 | |
|  |  |  |  | |
|  |  |  |  | |
|  |  |  |  | |
|  |  |  |  | |
|  |  |  |  | |
|  |  |  |  | |
|  |  |  |  | |
|  |  |  |  | |
|  |  |  |  | |

## **Figure S13**. GPC data of 10 wt% **PCPC-16** in **PCPC-76**.

|  |  |  |  | |
| --- | --- | --- | --- | --- |
|  | ***M*_n_**  **(kg mol^-1^)** | ***M*_w_**  **(kg mol^-1^)** | ***Đ*** |  |
|  |  |  |  | |
|  | 36.5 | 68.3 | 1.87 | |
|  |  |  |  | |
|  |  |  |  | |
|  |  |  |  | |
|  |  |  |  | |
|  |  |  |  | |
|  |  |  |  | |
|  |  |  |  | |
|  |  |  |  | |
|  |  |  |  | |

## **Figure S14**. GPC data of 30 wt% **PCPC-16** in **PCPC-76**.

|  |  |  |  | |
| --- | --- | --- | --- | --- |
|  | ***M*_n_**  **(kg mol^-1^)** | ***M*_w_**  **(kg mol^-1^)** | ***Đ*** |  |
|  |  |  |  | |
|  | 24.6 | 51.0 | 2.07 | |
|  |  |  |  | |
|  |  |  |  | |
|  |  |  |  | |
|  |  |  |  | |
|  |  |  |  | |
|  |  |  |  | |
|  |  |  |  | |
|  |  |  |  | |
|  |  |  |  | |

## **Figure S15**. GPC data of 50 wt% **PCPC-16** in **PCPC-76**.

## **Figure S16**. DSC data of monomodal PCPC series.

## **Figure S17**. TGA data of monomodal PCPC series.

## **Figure S18**. DSC data of narrow-gap bimodal PCPC series.

## **Figure S19**. TGA data of narrow-gap bimodal PCPC series.

## **Figure S20**. DSC data of **PCPC-9** blends in **PCPC-76**.

## **Figure S21**. DSC data of **PCPC-16** blends in **PCPC-76**.

## **Figure S22**. Wide-gap PCPC blend *T*_g_-s compared to the Fox equation applicable for miscible binary polymer blends.

The dashed lines, colour-coded with respect to the low-*M*_n_ PCPCs blended to **PCPC-76**, represent the trend predicted by the Fox equation (**Equation S3**) used to describe the *T*_g_ – blend fraction relationship in binary polymer blends.^3^ *T*_g_-s of the monomodal PCPCs used for blending (**Figure S16**) were used as *T*_g,1_ and *T*_g,2_.

## **Figure S23**. Complete tensile data for **PCPC-76**.

## **Figure S24**. Complete tensile data for **PCPC-89**.

## **Figure S25**. Complete tensile data for **PCPC-88**.

## **Figure S26**. Complete tensile data for **PCPC-71**.

## **Figure S27**. Complete tensile data for **PCPC-81**.

## **Table S3**. Tensile properties and *T*_g_-s of narrow-gap bimodal PCPCs, compared to previous reports of PCPC thermal-mechanical properties.

| **PCPC sample** | ***T*_g_**^[a]^ **(°C)** | ***σ*_max_**^[b]^ **(MPa)** | ***ε*_b_**^[c]^ **(%)** | ***E*_Y_**^[d]^ **(GPa)** | ***U*_t_**^[e]^ **(MJ m^-3^)** |
| --- | --- | --- | --- | --- | --- |
| **PCPC-76** | 87 | 57.2 ± 0.6 | 9.0 ± 0.5 | 1.30 ± 0.05 | 3.7 ± 0.3 |
| **PCPC-89** | 87 | 60.1 ± 0.5 | 7.7 ± 0.5 | 1.46 ± 0.02 | 3.2 ± 0.3 |
| **PCPC-88** | 88 | 61.0 ± 1.0 | 8.9 ± 0.5 | 1.27 ± 0.03 | 3.6 ± 0.3 |
| **PCPC-71** | 87 | 58.8 ± 0.6 | 7.3 ± 0.5 | 1.34 ± 0.03 | 2.8 ± 0.3 |
| **PCPC-81** | 88 | 61.1 ± 0.5 | 8.9 ± 0.4 | 1.36 ± 0.03 | 3.8 ± 0.2 |
| **PCPC-114^4^** | 85 | 58.5 ± 0.7 | 7.1 ± 0.8 | 1.70 ± 0.04 | 2.9 ± 0.4 |
| **PCPC-19^5^** | 70 | 20 | 34 | Not reported | Not reported |

Polymers were labelled corresponding to their molecular weight *M*_n_. ^[a]^determined by DSC. ^[b]^Maximum tensile strength, coinciding with yield stress in all cases except for **PCPC-19** where stress at break was reported.^5^ ^[c]^strain at break. ^[d]^Young’s modulus. ^[e]^Ultimate tensile toughness. Errors represent the standard error of the mean, see **Equation S1**.

## **Figure S28**. Complete tensile data for **PCPC-9** blends in **PCPC-76**.

## **Figure S29**. Complete tensile data for **PCPC-16** blends in **PCPC-76**.

**Figure S30**. Time-temperature superposition data constructed from frequency sweeps on **PCPC-76**, referenced at 140 °C. *G’* represents storage, *G”* represents loss modulus, *tan* *δ* = *G”*/*G’*. Frequency sweep data was collected at 0.1% strain, 0.01-100 Hz and temperatures from 100 °C to 200 °C with +10 °C intervals. See full dataset on **Figure S32**.

**Figure S31**. Shift factors used for the construction of the TTS curve of **PCPC-76**, fitted against the Williams-Landel-Ferry equation.

|  |  |
| --- | --- |

**Figure S32**. Complete dataset of storage and loss moduli obtained from oscillatory frequency sweep experiments on **PCPC-76** at 0.1% strain.

**Figure S33**. Time-temperature superposition data constructed from frequency sweeps on **PCPC-9**, referenced at 140 °C. *G’* represents storage, *G”* represents loss modulus, *tan δ* = *G”*/*G’*. Frequency sweep data was collected at 0.1% strain, 0.01-100 Hz and temperatures from 100 °C to 170 °C with +10 °C intervals. Measurements above 170 °C were unsuccessful due to high flow of the melt and thus excluded from the set. See full dataset on **Figure S34**.

**Figure S34**. Shift factors used for the construction of the TTS curve of **PCPC-9**, fitted against the Williams-Landel-Ferry equation.

|  |  |
| --- | --- |

**Figure S35**. Complete dataset of storage and loss moduli obtained from oscillatory frequency sweep experiments on **PCPC-9** at 0.1% strain.

## **Figure S36**. Oscillatory temperature ramp of **PCPC-76** (0.1% strain, 1.0 Hz, 3 °C min^−1^). Top: dynamic moduli, G’ and G’’. Bottom: tan(δ).

## **Figure S37**. Oscillatory temperature ramp of **PCPC-81** (0.1% strain, 1.0 Hz, 3 °C min^−1^). Top: dynamic moduli, G’ and G’’. Bottom: tan(δ).

## **Figure S38**. Complex viscosity of **PCPC-76** and **PCPC-81**, obtained from oscillatory temperature ramp experiments.

## **Figure S39**. Solid-state depolymerization of **PCPC-76** using LCo(II)Mg(II)(OAc)_2_ catalyst at 1:300 [catalyst]:[PCPC repeat unit] loading at 140 ˚C. The data between 80-20% mass loss is fit to exponentials to determine *k*_obs_.

**Figure S40**. Solid-state depolymerization of **PCPC-89** using [LCo(II)Mg(II)(OAc)_2_] catalyst at 1:300 [catalyst]:[PCPC repeat unit] loading at 140 ˚C. The data between 80-20% mass loss is fit to exponentials to determine *k*_obs_.

## **Figure S41**. Solid-state depolymerization of **PCPC-88** using LCo(II)Mg(II)(OAc)_2_ catalyst at 1:300 [catalyst]:[PCPC repeat unit] loading at 140 ˚C. The data between 80-20% mass loss is fit to exponentials to determine *k*_obs_.

**Figure S42**. Solid-state depolymerization of **PCPC-71** using LCo(II)Mg(II)(OAc)_2_ catalyst at 1:300 [catalyst]:[PCPC repeat unit] loading at 140 ˚C. The data between 80-20% mass loss is fit to exponentials to determine *k*_obs_.

## **Figure S43**. Solid-state depolymerization of **PCPC-81** using LCo(II)Mg(II)(OAc)_2_ catalyst at 1:300 [catalyst]:[PCPC repeat unit] loading at 140 ˚C. The data between 80-20% mass loss is fit to exponentials to determine *k*_obs_.

## **Table S4**. Depolymerization rates of narrow-gap bimodal PCPCs.

| **PCPC sample** | ***k*_obs_^[a]^ (h^-1^)** | ***R*^2^** | **TOF^[b]^ (h^-1^)** |
| --- | --- | --- | --- |
| **PCPC-76** | 14 | 0.9855 | 1900 |
| **PCPC-89** | 17 | 0.9838 | 2300 |
| **PCPC-88** | 18 | 0.9861 | 2400 |
| **PCPC-71** | 12 | 0.9892 | 1600 |
| **PCPC-81** | 15 | 0.9784 | 2000 |

^[a]^*k*_obs_ calculated from exponential fits between 20-80% conversion from PCPC mass loss vs time data. ^[b]^Turnover frequency: moles of PCPC repeat units consumed (20-80% conversion)/moles of catalyst/time.

## **Figure S44**. Representative vapour-phase TGA-FTIR analysis of solid-state PCPC depolymerization compared against FTIR spectra of CPO and CO_2_ monomer standards.

# References

(1) McGuire, T. M.; Deacy, A. C.; Buchard, A.; Williams, C. K. Solid-State Chemical Recycling of Polycarbonates to Epoxides and Carbon Dioxide Using a Heterodinuclear Mg(II)Co(II) Catalyst. *J. Am. Chem. Soc.* **2022**, *144* (40), 18444–18449. DOI: 10.1021/jacs.2c06937

(2) Fox, T. G.; Flory, P. J. Second-Order Transition Temperatures and Related Properties of Polystyrene. I. Influence of Molecular Weight. *J. Appl. Phys.* **1950**, *21* (6), 581–591. DOI: 10.1063/1.1699711

(3) Kalogeras, I. M.; Brostow, W. Glass transition temperatures in binary polymer blends. *J. Polym. Sci., Part B:Polym. Phys.* **2008**, *47* (1), 80–95. DOI: 10.1002/polb.21616

(4) Rosetto, G.; Vidal, F.; McGuire, T. M.; Kerr, R. W. F.; Williams, C. K. High Molar Mass Polycarbonates as Closed-Loop Recyclable Thermoplastics. *J. Am. Chem. Soc.* **2024**, *146* (12), 8381–8393. DOI: 10.1021/jacs.3c14170

(5) Yang, G. W.; Wang, Y.; Qi, H.; Zhang, Y. Y.; Zhu, X. F.; Lu, C.; Yang, L.; Wu, G. P. Highly Selective Preparation and Depolymerization of Chemically Recyclable Poly(cyclopentene carbonate) Enabled by Organoboron Catalysts. *Angew. Chem., Int. Ed.* **2022**, *61* (46), e202210243. DOI: 10.1002/anie.202210243
